# Proteomic Signature and mRNA Expression in Hippocampus of SAMP8 and SAMR1 Mice during Aging

**DOI:** 10.3390/ijms232315097

**Published:** 2022-12-01

**Authors:** Marcella Reale, Erica Costantini, Lisa Aielli, Fabrizio Di Giuseppe, Stefania Angelucci, Mohammad A. Kamal, Nigel H. Greig

**Affiliations:** 1 Department of Innovative Technologies in Medicine and Dentistry, G. d’Annunzio University of Chieti and Pescara, 66100 Chieti, Italy; 2 Department of Medicine and Sciences of Aging, G. d’Annunzio University of Chieti and Pescara, 66100 Chieti, Italy; 3 Center for Advanced Studies and Technologies (CAST), G. d’Annunzio University of Chieti and Pescara, 66100 Chieti, Italy; 4 Institutes for Systems Genetics, Frontiers Science Center for Disease-Related Molecular Network, West China Hospital, Sichuan University, Chengdu 610065, China; 5 King Fahd Medical Research Center, King Abdulaziz University, Jeddah 21589, Saudi Arabia; 6 Department of Pharmacy, Faculty of Allied Health Sciences, Daffodil International University, Dhaka 1341, Bangladesh; 7 Enzymoics, 7 Peterlee Place, Hebersham, NSW 2770, Australia; 8 Novel Global Community Educational Foundation, Hebersham, NSW 2770, Australia; 9 Drug Design and Development Section, Translational Gerontology Branch, Intramural Research Program, National Institute on Aging, National Institutes of Health, Baltimore, MD 20892, USA

**Keywords:** aging, cholinergic system, inflammation, cytokines, hippocampus

## Abstract

Aging is a complex process often accompanied by cognitive decline that represents a risk factor for many neurodegenerative disorders including Alzheimer’s and Parkinson’s disease. The molecular mechanisms involved in age-related cognitive decline are not yet fully understood, although increased neuroinflammation is considered to play a significant role. In this study, we characterized a proteomic view of the hippocampus of the senescence-accelerated mouse prone-8 (SAMP8), a model of enhanced senescence, in comparison with the senescence-accelerated-resistant mouse (SAMR1), a model of normal aging. We additionally investigated inflammatory cytokines and cholinergic components gene expression during aging in the mouse brain tissues. Proteomic data defined the expression of key proteins involved in metabolic and cellular processes in neuronal and glial cells of the hippocampus. Gene Ontology revealed that most of the differentially expressed proteins are involved in the cytoskeleton and cell motility regulation. Molecular analysis results showed that both inflammatory cytokines and cholinergic components are differentially expressed during aging, with a downward trend of cholinergic receptors and esterase enzymes expression, in contrast to an upward trend of inflammatory cytokines in the hippocampus of SAMP8. Together, our results support the important role of the cholinergic and cytokine systems in the aging of the murine brain.

## 1. Introduction

As aging is a biological phenomenon that can lead to a decline in the physiological functions of the brain, an increase in the number of elderly people in the world termed the “silver tsunami” highlights the present need to understand the processes that characterize early or potentially pathological aging. With increasing age, changes in brain morphology as well as levels of neurotransmitters and hormones have been observed [1,2]. These, in large part, correlate with cognitive decline, memory loss and a reduction in executive function. Aging is accompanied by expression changes in multiple genes and particularly in inflammatory genes, which are the most upregulated in the aging brain within several key regions [3,4,5]. Pro-inflammatory cytokines levels have been found to be greater in the brains of old-aged mice (22–24 months) as compared with adults (3–4 months) [6]. Additionally, peripheral markers of inflammation are elevated in aging people, and appear to act as a risk factor for the development or progression of age-related neurodegenerative disease [7]. Albeit that aging is the strongest risk factor for neurodegenerative disorders, and whereas aging is not considered a disease, neuroinflammation is one of the key factors that drives neurodegeneration in several brain diseases, as epitomized by Alzheimer’s disease (AD). Neuroinflammation compromises the maintenance of homeostasis as well as neuronal function in the aging brain [8] and likely drives cognitive and behavioral alterations by multiple mechanisms, among which are the regulation of gene expression, alterations in neuronal function and reduced neurogenesis [9,10,11]. In this light, a vicious circle is established, in which aging may amplify the effects of neuroinflammation on cognition, and inflammation can exacerbate the effects of aging on cognitive decline.

There is established evidence indicating deterioration of cholinergic function during brain aging [12,13,14,15] and a marked surge in neuroinflammation with senescence. According to the cholinergic hypothesis of AD, a correlation between changes in cholinergic function and cognitive decline has been proposed in dementia and aging. Support for this hypothesis is based on pharmacological research and on histological analysis of brain pathology in AD patients [16,17,18]. Acetylcholinesterase (AChE) and butyrylcholinesterase (BuChE) by inactivating acetylcholine (ACh) can potentially modulate inflammation [19,20,21] as evident in various clinical conditions [22,23,24]. Inhibition of the neuronal AChE enzyme augments ACh levels at the cholinergic synapses, partly counteracts the cholinergic loss and can ameliorate AD cognitive symptoms [25]. Shytle et al. proposed the presence of a “cholinergic anti-inflammatory pathway” (CAP) within the central nervous system (CNS) mediated by the activation of nicotinic acetylcholine receptors (nAChR) α7, whose dysfunction would tip the balance towards greater inflammation [26]. Thus, hallmarks of aging and neurodegenerative disorders can be represented by and associated with neuroinflammation and cholinergic dysfunction. The brain is organized into multiple areas with relatively specialized functions, and multiple inter-areal interactions generally underlie its various behaviors and cognitive functions. Function alterations can precede histopathological degeneration in aging brain, suggesting that analysis of brain gene expression may offer unique insights into the molecular mechanisms underlying age-related changes [27]. Thus, a breakdown in inter-regional coordination is likely an important pathophysiological mechanism that underpins neurodegenerative disorders such as Parkinson’s disease (PD) and AD [28,29]. 

In the hippocampus, aging is associated with neuronal deterioration, evidenced by a reduction of neuronal size and count [30], decrease of synaptic connections [31], intracellular pathology [32] and complexity, and plasticity [33], suggesting that the hippocampus is particularly exposed to the effects of aging. Although the mechanisms are not yet fully understood, increased neuroinflammation appears to have a key role in these age-related declines and reduced cholinergic function has been related to such increases in neuroinflammation and progressive memory deficits in both normal and accelerated aging, corresponding to deficits in hippocampal neuronal plasticity [13,34,35,36,37,38,39].

As indicated above, many age-related changes of inflammatory and cholinergic markers have been evaluated, but without highlighting a relationship between them. To understand whether there is an aging-associated inter-relationship between cholinergic and inflammatory changes within the hippocampus, we evaluated this in the spontaneous senescence-accelerated mouse prone-8 (SAMP8) model as it displays a phenotype of accelerated aging with memory, cognitive and behavioral impairments. This murine model is accompanied by molecular features typical of AD and is a valuable facsimile for obtaining more in-depth information on potential age-related neurodegenerative processes [15,40,41,42]. We additionally used the senescence-accelerated-resistant mouse (SAMR1), which has a similar genetic background and is extensively used as a control model as it shows normal aging characteristics [43]. 

In this study, we collected brain and hippocampus samples, as the latter is a particularly important brain area related to learning and memory. This allowed us to perform hippocampal and brain proteomics to define their protein profiling in both SAMP8 and SAMR1 animals, as well as to compare the hippocampus as a brain region involved in cognition and implicated in AD with a composite brain sample that combined multiple other brain regions. Additionally, to better understand alterations in hippocampal processes underlying aging, we integrated proteomic evaluation with gene expression profiles by quantitative Real-Time PCR (qPCR) carried out to evaluate the relationship between the expressions of nAChR cholinergic receptor subtypes with those of the cholinergic esterase enzymes and inflammatory cytokines, interleukin (IL)-1β and tumor necrosis factor (TNF)α, and to investigate the way in which gene expression levels change concurrently with aging.

## 2. Results

### 2.1. The 2DE Analysis of the Neuroproteome Changes by Comparison between the Cortical and Hippocampal Regions in SAMP8 and SAMR1

For each sample of 4-(T4) and 8-(T8)-month aged SAMR1 (R) and SAMP8 (P), brain cortex (BR-BP) and hippocampus the 2D proteomic analyzes were performed using a comparative approach. Each sample was analyzed in triplicate. In T4 month aged mice, a total amount of 5.8 mg in brain cortex of SAMR1 (BR), 6.2 mg in brain cortex of SAMP8 (BP), 3.3 mg in hippocampus of SAMR1 (HR) and 4.2 mg in hippocampus of SAMP8 (HP) of protein were extracted. In contrast, we obtained approximately 1.9 mg (HR) and 6.2 mg (HP) of protein from the T8 group. For each extracted sample, three 2D maps were produced, loading 150 μg of total proteins on a narrower pH gradient (4–7). The 2D electrophoretic run on gradient gel (9–16%) resolved 1321 ± 17 and 1394 ± 7 protein spots on the T4 analysis group for the cerebral cortex and hippocampus in SAMR1, respectively. The composite brain and hippocampus proteome in SAMP8 shows 1190 ± 8 and 1351 ± 6 protein spots, respectively, all distributed in a pH range 4–7. It is observed that the hippocampal areas of both models have a greater number of protein spots than the brain areas. On the contrary, for the T8 group in which only the hippocampal area was taken into consideration, the number of protein spots is 1223 ± 29 (HR) and 1274 ± 7 (HP) (Table 1).

In this preliminary proteomics step, we evaluated only molecular species such as specific tissue proteins to be analyzed in terms of their level of expression during brain aging. Two-dimensional analysis of the neuroproteomes allowed in the first and second comparison (1st and 2nd matching), between both the murine strains, SAMP8 and SAMR1, to identify the hippocampal proteins with the tissue-specific functional role and to have used them as a variable expression target. It was observed that the hippocampal areas of both models have a greater number of protein spots than the cortical areas. Another interesting finding is the different expression of proteins common to the two cerebral areas, brain and hippocampal, tissue-specific for the SAMR1 and SAMP8 models; those quantitative differences with a *p*-value < 0.05 were statistically significant, according to the one-tailed ANOVA test. 

### 2.2. Identification of Hippocampus-Specific Proteins in SAMR1 and SAMP8 Mice 

In SAMR1, 45 hippocampal proteins showed a low expression level, and 4 proteins showed a significantly high expression. In SAMP8, 11 proteins were characterized by a low expression level, and 9 proteins by high expression. The tissue-specific quantitative differences in the SAMR1 and SAMP8 models, identified by MS, are reported in Tables S1 and S2 (in Supplementary Materials). In the hippocampus of SAMR1 mice, a modest increase in expression was observed in the molecular complex associated with cytoskeletal actin, which is involved in regulation of the cell migration process. In SAMP8 mice, a higher level of expression was evident in proteins involved in energy metabolism, such as cytoplasmic malate dehydrogenase, MDHC (glycolysis) and phosphoglycerate mutase 1, PGAM1 (gluconeogenesis and pentose shunt). In addition, there are some up-regulated proteforms involved in signal transduction, such as a transmembrane protein, VATB2, an ATPase for the cellular transport and chemitin reductase, CRYM, an enzyme with a key role in the regulation of the biosynthesis of various neuropeptides (cystathionine-chemitin, CysK; lanthionine-chemitin, LK) as well as in the secretion of thyroid hormones and in the modulation of the intracellular concentration of T3. We also found the isoform 3 of a phosphatase, DUS3, strongly upregulated; this protein acts as a regulator of the cell biosynthesis process, a modulator of the MAPK, ERK1 and ERK2 kinase systems and of the T cell activation pathway, but, above all, it exerts a positive influence on cellular mitosis (cell proliferation). Among the proteins with a reduced level of expression in the hippocampal area, two isoforms of the stathmin protein (STMN) were identified for both models, the STMN1 with pI 5.6 and PM 17158 and the second with pI 5.3 and PM 17406, whose high specificity of binding with tubulin determines a role of primary importance in the developmental biology of the cerebral system, in particular in the formation of the axon, in cell proliferation (formation of the mitotic spindle) and in the more interesting role of a response to viral infections. Another proteomic phenotype common to the two models is the expression of two isoforms of type C superoxide dismutase (SODC), an enzyme involved in the removal of ROS physiologically released within the cell during oxidative metabolism. Among the constitutively expressed proteins of SAMP8, the pyridoxal kinase, PDXK, has been identified as important in the conversion of vitamin B6 (pyridoxine or pyridoxamine) into pyridoxal 5-phosphate (PLP), a ubiquitous cofactor for different enzymatic reactions in eukaryotic cells. Furthermore, comparing the cortical with hippocampal areas, for both murine strains, in order to confirm those model-specific quantitative differences correlated only to cerebral aging, five up-regulated proteins in the cortical area were identified and listed in Table 2. They include NADH dehydrogenase (NDUS3), cytochrome b5 (CYB5) and cytochrome c (COX5B), all located in the mitochondrial compartment as components of the respiratory chain. These five up-regulated proteins also include an integral membrane protein (ITM2B) involved in the inhibition of synthesis of the precursor of β-amyloid (Aβ) peptide, predisposing the fibrillar deposition of Aβ peptide in neurodegenerative syndromes, such as AD, and finally a ribosomal protein, ubiquitin-60S L40 (RL40). The RL40 protein, both as a component of the 60S subunit and in the form of polyubiquitin, covalently linked to the residues of Lys 6-11-29-33-48-63, plays a role in DNA repair, protein degradation-related to the REL and proteasome pathway, in lysosomal digestion, and controls function in protein biosynthesis (kinase pathway). It additionally has influence on signaling and cell cycle mechanisms. In the hippocampal area, among the differentially regulated proteins, chemitin reductase (CRYM) and phosphatase (DUS3) have been identified.

### 2.3. Effect of Aging on the Neuroproteome

By comparative study carried out to observe changes in the neuroproteome related only to brain aging between SAMP8 and SAMR1 mice, in the sample from T4 (i.e., at 4 months) vs. T8 (i.e., at 8 months), 14 up-regulated proteins were identified in the SAMR1 model, whereas in the SAMP8 model, 8 were found as differentially expressed proteins, of which 4 were up-regulated and 4 had a reduced level of expression. The SAMR1 hippocampus proteome includes seven proteins involved in the regulation of metabolic processes (Gene Ontology, http://geneontology.org (accessed on 3 February 2021)) such as inorganic pyrophosphatase (IPYR), the Ca2 + binding protein in the EF domain (helix-loop-helix) (EFHD2), calbindin 1 (CALB1), malate dehydrogenase (MDHC), two isoforms of regulatory phosphatase 1 (PP1R7) with same pI 4.9 and with MW of 33971 and 34021 Da, respectively, and dopachrome decarboxylase (DOPD). There are also two proteins belonging to the regulatory functional category of the cytoskeleton and cell motility, the acid isoform (pI 5.3) of stathmin (STMN1) and the long-chain fatty acid binding protein (FABPH), and two proteins of the category functional signal transmission and cellular transport, i.e., NSF-binding protein alpha (N-ethylmaleimide-sensitive factor) (SNAA) and the nuclear and transmembrane-like 3-like isoform of microfibril-associated protein d (MFA3L). Finally, there are annexin A5 (ANXA5), the acid isoform (pI 5.9) of Superoxide dismutase type C (SODC), belonging to the functional group of the cellular response under stimuli and the 40S ribosomal protein (RSSA) involved in the biosynthesis of nucleotides (Table 3).

In the SAMP8 model (Table 4), the up-regulated proteins, i.e., the acidic and basic isoforms of STMN1 (pI 5.3 and 5.6), an additional isoform of MDHC (pI 6.2) and Pyridoxal Kinase (PDXK) by GO analysis show the following percentage functional distribution: 50% in the regulatory category of the cytoskeleton and cell motility; 25% in the category of cellular metabolic processes; 25% in protein biosynthesis, folding and degradation. The proteins characterized by a reduced level of expression are functionally distributed in the following way: CRYM in the class of protein biosynthesis, folding and degradation (25%); PGAM1 in the cell metabolism class (25%); VATB2 in the cellular transport category (25%); the SODC isoform (pI 6.02) in the cellular response under stimuli (25%).

### 2.4. Bioinformatics Analysis of Neuroproteome Differences in Aging-Related Expression

**Figure 1:**
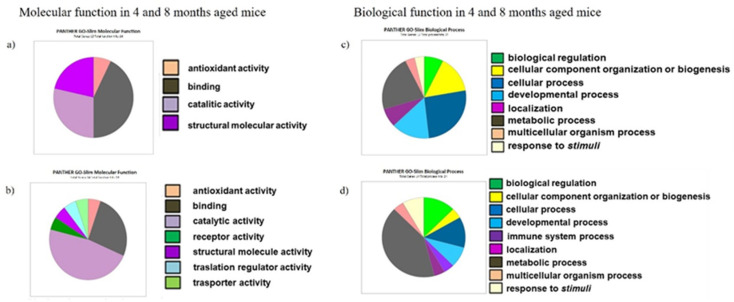
PANTHER classification by Gene Ontology of the common and specific proteins in SAMP8 and SAMR1 at 4 and 8 months; (**a**) molecular function distribution in 4-month-aged mice; (**b**) molecular function distribution in 8-month-aged mice; (**c**) biological process classification in 4-month-aged mice; (**d**) biological process classification in 8-month-aged mice.

To fully understand the molecular function, the biological process and the cellular distribution of proteins modulated in their expression by the brain aging process, we imported the data produced by the identification analysis using mass spectrometry (MS) into the GO and PANTHER database. This classification system organized the different proteforms into four categories (SAMR1 and SAMP8, at 4 months) and into seven categories (SAMR1 and SAMP8 A, at 8 months) based on molecular function (Figure 1a,b), and into eight (SAMR1 and SAMP8, 4 months) and nine categories (SAMR1 and SAMP8 A, at 8 months) by biological process (Figure 1c,d).

### 2.5. Cytokines and Cholinergic Marker Expressions in Brain 

In light of the broad role of inflammatory cytokines in neurodegeneration, including their involvement in synaptic plasticity, oxidative, metabolic, and excitotoxic stress, to study whether transcriptional changes occur during aging we analyzed brain transcripts of TNFα and IL-1β in 4- and 8-month-aged SAMP8 in comparison to SAMR1 aged-matched mice. A higher expression of IL-1β and TNFα was detected in both 4- and 8-month-aged SAMP8 with respect to SAMR1 mice of the same age (Table 5). The evaluation of cholinergic marker gene expressions showed a reduction in 8-month-aged SAMP8 mice of BuChE, AChE and nAChRα7, although not significant. Moreover, the nAChRα4 and nAChRβ2 gene expression showed not significant variations in the brain of SAMP8 in comparison with SAMR1 age-matched mice (Table 5). 

### 2.6. Cytokines and Cholinergic Marker Expressions in Hippocampus

A significant up-regulation of both TNFα and IL-1β was similarly detected in the hippocampus of 4- and 8-month-aged SAMP8 mice with respect to SAMR1 mice (Table 6). An evaluation of time dependency demonstrated a mild rise in IL-1β (+9%) and TNFα (+12%), respectively, in 8-month with comparison to 4-month SAMP8 mice.

On comparing AChE and BuChE expressions in the hippocampus of SAMP8 and SAMR1 mice, a substantial and significantly higher expression of BuChE was detected in 4- and 8-month-aged SAMP8 mice with respect to SAMR1 mice. In contrast, a weakly higher expression of AChE that did not reach statistical significance was evident in the hippocampus of 8-month SAMP8 versus SAMR1 mice. Additionally, an increase in the expression of AChE and BuChE was observed at +35% and +14%, respectively. A greater expression of nAChRs α4, α7 and β2 subunits, with respect to SAMR1 mice, was found in SAMP8 hippocampus with a higher rise in α4 (*p* < 0.01) in 8-month mice and of α7 (*p* < 0.05, <0001) in 4- and 8-month-aged SAMP8 mice, respectively. A time-dependent increase in hippocampal expression levels of α4 (+100%) and of β2 (+23%) subunits was determined between 4 and 8 months of age in SAMP8 mice, whereas observed levels of the α7 subunit remained relatively unchanged (+3%).

### 2.7. Differential Expression of Cytokines and Cholinergic Markers in Hippocampus and Brain

When comparing the TNFα and IL-1β expression levels in the hippocampus vs. those of composite brain in 4- and 8-month-aged SAMP8 mice, a significantly higher expression was observed in the hippocampus for both inflammatory cytokines (Table 7).

The BuChE and AChE expression levels in the hippocampus of 4-month-aged SAMP8 mice were +1.58-(*p* < 0.05) and +1.08-fold higher than those in the composite brain sample and in 8-month animals +2.38-(*p* < 0.001) and +1.84-fold higher (*p* < 0.01), respectively. The expression levels of nAChRα7 in the hippocampus were approximately +4 (*p* < 0.001) and +8-fold higher than in the composite brain, respectively, in 4- and 8-month SAMP8 mice. In contrast, nAChRα4 expression was lower in the hippocampus than in the composite brain sample in both 4- and 8-month SAMP8 mice, whereas β2 expression was higher in the hippocampus than in brain, reaching statistical significance in SAMP8 8-month-aged mice (Table 7). 

## 3. Discussion

The hippocampus is a brain area fundamental to cognition, which possesses a key role in learning and memory, and previous studies have revealed that hippocampal aging is largely similar in humans and in animal models [44,45]. During aging, a cholinergic hypofunction, related to progressive memory impairment, has been detected. Normal aging is accompanied by a gradual loss of cholinergic function caused by dendritic, synaptic, and axonal loss as well as a decrease in trophic support, whereas pathological aging has been characterized by neuronal cell loss. Age-related losses of hippocampal-dependent memory may be due to altered synaptic plasticity mechanisms, including long-term potentiation (LTP) that can lead to the dysregulation of inflammatory pathways, neurogenesis, and ultimately to apoptosis [46,47,48]. To date, interactome mapping and network-based examinations of gene-specific targets, with functional significance in hippocampal aging, have not yet been fully explored. 

Based on proteomic evaluation, we have characterized the molecular function, biological process and cellular distribution of hippocampal proteins that may be involved in early or pathological aging. We compared these to proteins deriving from a composite (i.e., combined) brain sample containing multiple brain regions, but not hippocampus, to highlight potential key differentially expressed proteins, and evaluated SAMP8 and SAMR1 mice on an alike genetic background as the former displays a phenotype of accelerated aging with ensuing memory, cognitive and behavioral impairments by 6 to 8 months of age [40,41,43]. 

The Protein Atlas database confirmed a strong expression of proteins was involved in metabolic and cellular processes in neuronal and glial cells of the hippocampus. Functional analysis of our data with the PANTHER database revealed that most of the differentially expressed proteins are involved in metabolic processes important for energy generation, apoptosis, cellular transport, organization of the cytoskeleton and cellular response, thereby highlighting the various molecular mechanisms involved in learning and memory capacity under the influence of the brain aging process in accordance with other studies that highlighted the role of metabolic alterations during aging [49,50,51]. In this regard, in hippocampus of SAMP8 and SAMR1 proteoforms, we found different regulations of proteins involved in the cytoskeleton and cell motility, metabolism, protein biosynthesis and cell response, signal transmission and cellular transport, suggesting a characteristic aging onset.

Sustained release of inflammatory cytokines may promote cognitive aging and diminished memory. Increased IL-1α levels in the hippocampus may be a trigger for impairments in LTP in age- and stress-induced rats [52]. The heightened secretion of TNFα which can alter astrocyte-neuron signaling and the excitability of hippocampal synapses and promote an increased possibility of engulfment of synapses by activated microglia [53] could result from a suppressed cholinergic anti-inflammatory pathway. 

The nAChRs together with cholinesterase activity, the status of the cholinergic anti-inflammatory pathway, and inflammatory cytokines are important factors in the development of neurodegenerative disorders such as AD, and hence their dysfunction has been extensively studied across several animal models [54,55]. It is known that memory deficits in both normal and accelerated aging are characterized by a reduction of the intrinsic neuronal plasticity of the hippocampus, and it has been hypothesized that changes in the expression of ion channels and/or receptors that perform a key role in the regulation of neuronal excitability and plasticity may provide a basis for the onset of memory deficits [56]. In this context, nAChRs have key roles in development and synaptic plasticity, and alterations in nicotinic mechanisms that participate in learning, memory and attention contribute to a variety of neurological disorders and diseases [57,58]. Several lines of evidence have led to the idea that there is a relationship between the activation of nAChRs and the maintenance of cognitive function in aging [59,60]. Aberrant alterations in muscarinic and nicotinic receptor expression may hence contribute to cholinergic dysfunction [61]. Among neuronal nAChRs, receptor proteins that form ligand-gated ion channels in the plasma membranes of neurons, α4-β2 and α7 are broadly expressed in the human brain and are involved in cognitive functions such as attention, learning, and memory [62]. In this regard, the hippocampus is a region with a high density of nAChRs as well as microglia and astrocytes [63], and the cholinergic stimulation of the hippocampus not only has direct neuronal effects, but also affects the microglia and astrocytes that may modulate neuronal function. Cognitive dysfunction caused by an interruption of the cholinergic pathway can potentially be ameliorated by activation of nAChRs present within the hippocampus, and since ACh lowers the release of inflammatory cytokines, an inhibition of cholinesterase activity modulates ACh hydrolysis, augmenting its level and thereby implementing its impact on the cholinergic blockade of inflammatory processes [64]. The activation of α7 is known to alter the phenotype of both macrophages and microglia from a pro-inflammatory macrophage (M)1-like to a more quiescent M2-like phenotype [65,66]. Consequently, any dysfunction in nAChRsα7 and related signaling processes could tip the balance towards more inflammation. However, ion channels and receptors are under-represented in many proteomic studies due to the heterogeneous and sparse nature of these proteins, especially when compared to highly abundant mitochondrial and cytosolic proteins [67]. To overcome this bias, we performed a qPCR, which allowed us to highlight a decreasing trend of β2 nAChR subtype expression in brain of both SAMR1 and SAMP8 mice, with a more significant variation in SAMP8 mice. The nAChR α4 expression was higher in brain tissue of 4-month-aged SAMP8 and not significantly different between SAMP8 and SAMR1 brains at 8 months; however, it spiked in the hippocampus of 8-month SAMP8 mice. Our results agree with the study of Tohgi et al., which showed an unaltered expression of α4 subunit mRNA and a significantly decreased level of β2 subunit mRNA expression in cortex and hippocampus in normal human aging [68].

The α7 subunits are likely expressed as homomeric assemblies that, when combined with heteromeric α4β2 assemblies, constitute the two major nAChR subtypes expressed in brain. We report the time-dependent up-regulation of the expression of the nAChRα7 subunit mRNA in hippocampus of both SAMR1 and SAMP8 mice with a higher expression in SAMP8 mice. Furthermore, significant differences were detected between hippocampus compared with composite brain, with higher nAChRα7 expression in 4-month-aged and higher nAChRb2 in 8-month-aged SAMP8 mice hippocampus. This suggests that, in SAMP8 mice, an increased nAChRα7 expression in hippocampus could stimulate auto receptor-mediated ACh release to augment the declining cholinergic signal as well as to maintain a stable presence of α7 in the hippocampus and compensate for its decrease in other brain areas. Our data are in accordance with Counts et al., who reported that in hippocampus there was little or no decrease in high-affinity nicotine binding during aging, whereas in the entorhinal cortex and in the presubiculum, a major loss of high-affinity nicotine binding was detected [69]. In human, a statistically significant association has been found between increasing nAChRα7 mRNA levels and a higher likelihood of AD by the National Institute on Aging and the Reagan Institute Working Group criteria [69]. The nAChRα7 subtype is widely considered to participate in several mechanisms of neuroprotection [70,71], such as in nicotine-mediated neuroprotection against either glutamate excitotoxicity or Aβ peptide challenge [72,73], and by blocking the release of pro-inflammatory cytokines [74]. 

The greater availability of cholinergic receptors is correlated with an increase in available binding sites and to greater receptor activation. Activation of cholinergic receptors triggers multiple pathways that cause post-translational modifications (PTMs) in multiple proteins to ultimately bring about changes within the nervous system. PTMs mediated by cholinergic receptors, in part, substantially influence the biosynthesis, proteolysis, degradation, and expression of many proteins. Our results showed a similar time-dependent increasing trend for IL-1β and TNFα expression, in line with a study that demonstrated that pro-inflammatory cytokines could influence the outcome of nicotinic receptor assembly and that cytokines modulating the assembly of receptors can influence the response of key neurotransmitter receptors [75].

Previous studies of the cholinergic system in aging SAMP8 and SAMR1 mice have produced inconsistent results with increases, decreases or no changes reported in pre- and post-synaptic cholinergic parameters [76,77]. A decreased choline acetyltransferase (ChAT) activity was reported in two subregions of the hippocampus of SAMP8, but not in the SAMR1 mice, by Strong et al., 2003, and during aging the spontaneous release of [3H]ACh was not significantly different in SAMP8 and SAMR1 mice [14,78]. Keeping this in mind, we focused our attention on the expression of pro-inflammatory cytokines and several key cholinergic system components. The CNS is a privileged immunological site, and the inflammatory mediators are relatively low in abundance, and thus, to guarantee high sensitivity, we carefully established and optimized our qPCR settings and were able to show that expression levels of all the evaluated inflammatory cytokines are increased with age. 

Other characteristics shared by our SAMP8 mice are the non-significant differences of AChE and BuChE expression in the brain samples between 4 and 8-month-aged SAMP8 and SAMR1 mice. In the hippocampus of 4- and 8-month-aged SAMP8 mice, a significantly higher expression of BuChE was detected with respect to aged-matched SAMR1 mice. These results are partially in accordance with results of Fernández-Gómez et al. that suggested a senescence-induced up-regulation of BuChE activity and invariable expression levels in SAMP8 brains [15]. 

A recently proposed role for BuChE as a compensatory mechanism for AChE activity, should it become insufficient, might explain the significant increases of BuChE in contrast to no variation of AChE in hippocampus of SAMP8 with respect to SAMR1 mice, as BuChE has been proposed as a co-regulator of cholinergic transmission in neurons when strongly stimulated and exposed to high amounts of ACh [25,79] or in a pathological setting as occurs in AD [80,81].

In the brain and hippocampus of SAMP8 mice, the mRNA expression of cholinergic markers was down-regulated over time; to the contrary, a higher expression was detected in the hippocampus of both 4- and 8-month-aged SAMP8 with respect to SAMR1 mice. Proteomic analysis did not highlight the presence of translated protein of cholinergic markers. This absence of correlation between mRNA expression and protein levels did not surprise us. Indeed, several studies have shown a poor or absent correlation between expression levels of protein and mRNA in mammals, and multiple factors such as post-transcriptional modifications of mRNA or protein stability that are not mutually exclusive could explain such poor correlations [82,83,84]. 

Among numerous factors, translational efficiency for mRNA may be modified by methylation, and actions of miRNA and post-transcriptional modifications of mRNA may be the reasons for low protein with respect to the mRNA level in a tissue [85]. Protein stability is another factor, as proteins may differ substantially in their in vivo half-lives. Whereas some proteins have a long half-life, others must be immediately destroyed or modified to support their time-sensitive function. Protein damage/degradation by reactive oxygen species (ROS) may contribute more in relation to the reverse result, and, in the brain of animal models, the evaluation of protein expression is complicated by the relatively low and often very heterogeneous expression within tissues of mixed cellular composition. 

The complexity of the human genome is enhanced by alternative splicing. This certainly applies to AChE mRNA that is susceptible to alternative splicing [86], and this leads to three post-transcriptional species that derive from the same gene [87]. In human brain and leukocytes, six mRNA splice variants of the α7 gene have been identified, although it remains unclear whether several of these transcripts are processed to functional protein [88]. Indeed, not all splice variants can be translated, and others cannot be readily quantified as they are of a different size, but analysis of large-scale mRNA and protein isoforms expression is beyond the aim of this study and can potentially be performed by proteogenomic assays.

Our results provide evidence that SAMP8 mice have changes in their cholinergic system from 4 to 8 months of age and an increase in pro-inflammatory cytokines expression, in line with reported declines in their cognitive capacity at 4 to 8 months of age [40,41,43]. 

More detailed future studies of the way in which each of these sets of molecules affects the local network of the hippocampus could potentially augment our understanding of the onset of neurodegenerative diseases and the way these differ from normal aging. 

## 4. Materials and Methods

### 4.1. Animals

Female SAMP8 and SAMR1 mice obtained from Charles River Laboratories were used in our study. Littermates were maintained under the same conditions. Mice were randomly divided into groups: 4-month-aged (SAMP8 n = 15; SAMR1 n = 15) and 8-month-aged (SAMP8 n = 15; SAMR1 n = 15) and housed in groups of 4 mice in ventilated racks, at 20–22 °C, under a 12 h day/night cycle with a half-hour transition at sunrise and sunset. All mice had ad libitum access to water (irradiated global diet 2918 Harlan and water autoclaved) and food (standard rodent diet) within the University of “G.d’Annunzio”, Chieti-Pescara Animal House Facility. Experimental procedures were conducted according to the European Communities Council Directive of 22 September 2010, for care of laboratory animals and after approval of the Local Ethics Committee of the University of “G.d’Annunzio”, Chieti-Pescara (PROG/48). All efforts were made to minimize the number of animals used in accordance with the ARRIVE guidelines and recommendations. 

Throughout performed experiments and resulting analyses, there were no exclusions of animals or data points. Groups of SAMP8 and SAMR1 mice were deeply anesthetized with pentobarbital (30–50 mg/kg) and euthanized by cervical dislocation at 4 and 8 months of age. The hippocampal area was dissected out of the brains. For proteomic analysis, all samples were frozen in liquid nitrogen and stored at −80 °C until further processing. For gene expression analysis, brains were removed, and hippocampus were rapidly dissected and removed on wet ice. The samples were stored in RNA-later at 4 °C for no more than two weeks, representing a time necessary to organize the workflow in the laboratory. Subsequently, we proceeded with the extraction of the RNA from the tissues and then conserved RNA samples at −80 °C. 

### 4.2. 2-DE Analysis

All comparative proteomics analyses were carried out on single samples in technical triplicate (each animal at 4 and 8 months of age) in order to analyze neuronal protein levels during aging. The hippocampus and brain tissue samples were individually homogenized and the cytosolic proteins were extracted with lysis buffer containing 7M urea, 2M thiourea, CHAPS and supplemented with tributyl-phosphine 2 mM and protease inhibitors (GE Healthcare, Uppsala, SE, USA). Protein concentration was measured using Pierce™ Better Bradford Assay (Thermo Fisher Scientific, Waltham, MA, USA) and total amounts of 150 µg (for the analytical gels) and 500 µg (for preparative gels) mixed with rehydration solution (DeStreak Rehydration Solution, GE Healthcare, Uppsala, SE, USA) were evaluated. Both were applied to isoelectric focusing (IEF) using IPG strip nonlinear pH 4–7, 24 cm (GE Healthcare, Uppsala, SE, USA) on the Ettan IPGphor III System (GE Healthcare, Uppsala, SE, USA). The second dimension Sodium Dodecyl Sulphate-PolyAcrylamide Gel Electrophoresis (SDS-PAGE) was performed on 12% SDS-PAGE according to procedures previously described by Angelucci et al. [89]. After staining, gels were scanned at 300 dpi with a GS-900 calibrated densitometer (Bio-Rad Laboratories, Hercules, CA, USA). A reference gel was created from a representative gel combining all spots common to each analyzed time point. The reference gel was then used to evaluate the presence of differences in protein expression among the matching conditions. Background subtraction was performed, and the intensity volume of each spot was normalized with total intensity volume (summing the intensity volumes obtained from all spots within the same 2-D gel). All the quantitative data are reported as mean ± SEM. The intensity volumes of individual spots were matched across the different gels and then compared among groups by multiple comparisons using one-way analysis of variance (ANOVA). A probability (*p*) value < 0.05 was considered statistically noteworthy. Significantly different protein spots were subjected to in-gel tryptic digestion and identification by mass spectrometry (MS).

### 4.3. Protein Digestion and MALDI TOF-TOF MS Analysis

All the protein spots whose intensity levels significantly differed among groups were excised from 2-D gels and were analyzed by using a peptide mass fingerprinting (PMF) approach with a MALDI-TOF/TOF spectrometer. A protein spot picked from a gel was washed with 100% ethanol and 100 mM ammonium bicarbonate ((NH4)HCO3). Then, the piece of gel was incubated for 60 min at 56 °C in 100 µL of 50 mM (NH4)HCO3 supplemented with 10 mM Dithiothreitol (DTT) and, thereafter, for 30 min in the dark in 100 µL of 50 mM (NH4)HCO3 supplemented with iodoacetamide at room temperature (RT). Ultimately, the gel was reswollen in 50 mM (NH4)HCO3 containing trypsin and was then incubated at 37 °C overnight [90]. A Peptide extract was applied to a C18ZipTip (Millipore, Bedford, MA, USA), rinsed with a 0.1% TFA and eluted directly on the MALDI target with 0.5 μL of a saturated α-cyano-4-hydroxycinnamic acid (1:1, ACN: 0.1% TFA) solution. Tryptic digests were analyzed by Autoflex Speed mass spectrometer (Bruker Daltonics, Bremen, DE) equipped with a Nd: YAG laser (355 nm, 1000 Hz) operated by FlexControl v3.3 and outfitted with a 355 nm nitrogen laser. All spectra were obtained with the delayed extraction technology in positive reflectron mode and averaged from 100 laser shots to improve the signal-to-noise (S/N) ratio. The voltage parameters were set at IS1 19 kV, IS2 16.7 kV, lens 8.5 kV, reflector 1 21.0 kV, and reflector 2 9.7 kV. The delay time was 10 ns, and the acquisition mass-to-charge range was 500–4000 Th. External high precision calibration (HPC) was performed using a peptide mixture containing bradykinin (fragment 1–7) 757.39 m/z, angiotensin II 1046.54 m/z, ACTH (fragment 18–39) 2465.9 m/z, Glu Fibrinopeptide B 1571.57 m/z, and renin substrate tetradecapeptide porcine 1760.02 m/z. Internal mass calibration was performed using trypsin autodigestion products (843.50 m/z, 1046.56 m/z, 2212.11 m/z, 2284.19 m/z). Samples analyzed by PMF were additionally analyzed using LIFT MS/MS from the same target. The most abundant ions per sample were chosen for tandem mass spectrometry (MS/MS) analysis. Analyses were performed in positive LIFT reflectron mode. The Precursor Ion Selector (PCIS) range was 0.65% of the parent ion mass. The voltage parameters were set at IS1 6 kV, IS2 5.3 kV, lens 3.00 kV, reflector 1 27.0 kV, reflector 2 11.45 kV, LIFT1 19 kV and LIFT2 4.40 kV. Each tryptic digest produced a spectrum in the PMF mode with a m/z range 700–3000b Da that was calibrated with internal mass standards from the trypsin autodigestion products (843.50 m/z, 1046.56 m/z, 2212.11 m/z, 2284.19 m/z) and subtracted from the peaks of trypsin and keratin contaminants. PMF data was entered into a database (NCBI and Swiss Prot) through the Mascot search engine, and this allowed us to compare the experimental masses from tryptic digest with those theoretical for all selected protein spots in accordance with the following research parameters: peptide mass fingerprinting, trypsin, fixed modifications such as carbamidomethylation (Cys), variable changes such as oxidation of methionine, monoisotopic mass, charge peptide state +1, miss cleavage up to 1, and mass tolerance for each peptide at 100 ppm. Next, all assigned proteins were validated by LIFT MS/MS technology after selecting a maximum number of 4 precursor ions per samples to be subjected to MS/MS analysis. The database search through Mascot was based on the use of combined PMF and MS/MS data using the BioTools 3.2 program connected to the Mascot search engine. The probability score that corresponds to a matching between the experimental data and each sequence deposited in the database with *p* < 0.05 was used as a criterion for correct identification. The scores were reported as log10 (P), where P represents the maximum probability. The acceptable score value was set at 70 for PMF and 30/40 for the MS/MS research.

### 4.4. RNA Extraction, Reverse Transcription, and Real-Time PCR 

Brain tissues were removed from RNAlater and total RNA from the SAMP8 and SAMR1 mouse brain was isolated using the QIAzol lysis reagent (Qiagen, Hilden, Germany), in accordance with the manufacturer’s recommendations. As previously described by Reale et al. [91], the concentration of total RNA was assessed with a NanoDrop 2000 UV-Vis Spectrophotometer (Thermo Scientific, Waltham, MA, USA). Thereafter, 1 µg of total RNA was transcribed to cDNA using the QuantiTec Revers Transcription Kit with integrated removal of genomic DNA contamination (Qiagen, Hilden, Germany) according to the manufacturer’s instructions. Next, q-PCR assays were performed in triplicate using GoTaq qPCR Master Mix (Promega, Madison, WI, USA), and specific mouse primer pairs were used to evaluate the expression of pro-inflammatory cytokines, nAChRs and cholinesterase enzymes (AChE and BuChE). Relative expression of each gene was normalized by use of the Hypoxanthine phosphoribosyltransferase (HPRT) gene by employing the ΔCt method, where ΔCt = Ct (BuChE, AChE, nAChR α7, α4, β2, IL-1β, TNFα)-Ct (HPRT). Primer pair sequences used in the study are reported in Table 8.

### 4.5. Bioinformatic Analysis

To understand the molecular function, biological process and cellular distribution of neuronal proteins expressed unequivocally in hippocampus from SAMR1 and SAMP8 mice that were then potentially changed in their content during brain aging, the data produced by the MS identification were imported into Protein Analysis Through Evolutionary Relationship (PANTHER) (http://www.pantherdb.org/ (accessed on 3 February 2021), SRI International, Menlo Park, CA, USA) and Gene Ontology (GO) databases [92]. 

### 4.6. Statistical Analysis

The reference gel was used to evaluate the presence of and difference in protein expressions. Background subtraction was performed, and the intensity volume of each spot was normalized by total intensity volume (summing the intensity volumes obtained from all spots within the same 2-D gel). All the quantitative data are reported as mean ± SEM values. The intensity volumes of individual spots were matched across the different gels and then compared among groups by multiple comparisons using one-way analysis of variance (ANOVA). A probability (*p*) value < 0.05 was considered statistically noteworthy. Significantly different protein spots were subjected to in-gel tryptic digestion and identification by mass spectrometry (MS). For qPCR calculated with the 2^−ΔΔCt^ method, gene expression levels are reported as mean ± SEM. Statistical comparisons between values from different treatments in the same model were calculated by GraphPad Prism software (http://www.graphpad.com) (accessed on 5 May 2021) using a Student *t*-test for unpaired data. *p*-values were corrected for multiple comparisons when appropriate.

## 5. Conclusions

Taken together, the proteomic profile of the hippocampus in SAMP8 and SAMR1 aged mice highlight those proteins functionally clustered in association with altered synaptic plasticity, oxidative stress, energy production and glutamate metabolism. Moreover, the age-related changes in gene expression linked to the inflammatory and cholinergic systems were detected. Although our preliminary study focused on proteomic profiling and mRNA expression evaluation, it provides interesting avenues for further research. Indeed, our results indicate that, in hippocampus of SAMP8 and SAMR1 mice, proteomic profiles and gene expressions can be affected differently during aging and thereby emphasize their importance in the development of effective therapies for the prevention and treatment of age-related cognitive decline.

## Figures and Tables

**Table 1:** Total spot number on 2D maps for T4 and T8 samples.

Murine Model (4 to 8 Months)	2D Gel CODE	Total Spot Number
***T4 ^a^***		
*SAMR1*	RH	1394 ± 7
	RB	1321 ± 17
*SAMP8*	PH	1351 ± 6
	PB	1190 ± 8
***T8 ^b^***		
*SAMR1*	SRH	1223 ± 29
*SAMP8*	SPH	1274 ± 7

*^a,b^* SAMR1 and SAMP8 aged 4 (T4) and 8 months (T8).

**Table 2:** MS-identified hippocampal proteins in SAMR1 and SAMP8.

	Protein Name	Abbreviation	AC	Theoretical Mr_pI	Experimental Mr_pI	*p*	PMF/ MS_2_	FV
Down-regulated	NADH dehydrogenase [ubiquinone] iron-sulfur protein 3, mitochondrial	NDUS3	Q9DCT2	30302_6.67	23351_5.6	8.9 × 10^−4^	12/3	0.7 ± 0.3
	Cytochrome c oxidase subunit 5B, mitochondrial	COX5B	P19536	14089_8.69	14686_5.8	0.002	10/1	1.2 ± 0.1
	Cytochrome b5	CYB5	P56395	15232_4.96	16409_4.8	0.010	7/1	0.2 ± 0.05
	Integral membrane protein 2B	ITM2B	O89051	30754_5.14	26241_5.05	1.8 × 10^−4^	9/-	1.3 ± 0.09
	Ubiquitin-60S L40	RL40	P62984	8033_6.89	12845_5.8	0.008	7/3	1.3 ± 0.01

AC: accession number, to identify the protein from SWISS PROT database; FV: fold variation, expression level degree. Mr2: relative molecular mass; PMF/MS2: Peptide mass fingerprint/Mass spectrometry. Statistical significance: *p* < 0.001.

**Table 3:** Neuroproteome changes in 8-month-aged SAMR1.

Protein	T8 (SAMR1)	FV
STMN1 ^1^	*up-regulated*	1.7 ± 0.01
FABPH ^1^	*up-regulated*	0.8 ± 0.18
IPYR ^2^	*up-regulated*	2.01 ± 0.2
EFHD2 ^2^	*up-regulated*	1.3 ± 0.05
CALB1 ^2^	*up-regulated*	0.7 ± 0.4
PP1R7 (MW: 33,971 Da) ^2^	*up-regulated*	0.9 ± 0.1
PP1R7 (MW: 34,021 Da) ^2^	*up-regulated*	0.4 ± 0.2
MDHC ^2^	*up-regulated*	2.1 ± 0.21
DOPD ^2^	*up-regulated*	0.8 ± 0.03
MFA3L ^5^	*up-regulated*	1.1 ± 0.16
ANXA5 ^6^	*up-regulated*	0.26 ± 0.8
SODC ^6^	*up-regulated*	0.8 ± 0.14
RSSA ^4^	*up-regulated*	1.9 ±0.02
SNAA ^5−7^	*up-regulated*	0.95 ± 0.1

Gene Ontology (GO) functional classification: ^1^: Cytoskeleton and cell motility; ^2^: Metabolism; ^3^: Protein Biosynthesis; ^4^: Nucleotide biosynthesis; ^5^: Signal transduction; ^6^: Cell response; ^7^: Trafficking. STMN: Stathmin; FABPH: Fatty acid binding protein heart; IPYR: Inorganic pyrophosphatase; EFHD2: EF-hand domain-containing D2; CALB1: Calbindin OS; PP1R7: Protein phosphatase 1 regulator subunit 7; MDHC: Malate dehydrogenase cytoplasmatic; DOPD: Dopachrome decarboxylase; MFA3L: Microfibrillar-associated protein 3-like; ANXA5: Annexin 5; SODC: Superoxide dismutase; RSSA:40S ribosomal protein SA; SNAA: Alpha-soluble NSF attachment protein. Table reports the FV: fold variation of each protein.

**Table 4:** Neuroproteome changes in 8-month-aged SAMP8.

Protein	T8 (SAMP8)	FV
CRYM ^3^	*down-regulated*	0.4 ± 0.2
PDXK ^3^	*up-regulated*	1.05 ± 0.01
MDHC ^2^	*up-regulated*	1.2 ± 0.05
PGAM1 ^2^	*down-regulated*	0.13 ± 0.8
VATB2 ^7^	*down-regulated*	1.7 ± 0.05
DUSP3 ^2,3^	*unchanged*	---
STMN1 ^1^	*up-regulated*	0.85 ± 0.11
SODC ^6^	*down-regulated*	0.8 ± 0.03

Gene Ontology (GO) functional classification: ^1^: Cytoskeleton and cell motility; ^2^: Metabolism; ^3^: Protein Biosynthesis; ^6^: Cell response; ^7^: Trafficking. CRYM: Kemitine reductase mu-crystallin; PDXK: Pyrodoxal kinase; MDHC: Malate dehydrogenase cytoplasmatic; PGAM1: Phosphoglycerate mutase 1; VATB2: V-type proton ATPase subunit B, brain isoform; DUSP: Dual specificity protein phosphatase 3; STMN: Stathmin; SODC: Superoxide dismutase. Table report the FV: fold variation of each protein.

**Table 5:** Gene expression (2^−ΔΔCt^) of IL-1β, TNFα, BuChE, AChE, nAChRα7, nAChRα4 and nAChRβ2 in the brain of 4- and 8-month-aged SAMP8 mice in relation to the brain samples from the age-matched SAMR1 mice.

	4-Month-Aged SAMP8	8-Month-Aged SAMP8
IL-1β	2.66 ± 0.38	2.39 ± 0.15 **
TNFα	1.18 ± 0.03 ***	1.40 ± 0.13 ***
BuChE	1.66 ± 0.15	0.70 ± 0.09
AChE	1.61 ± 0.15	0.72 ± 0.09
nAChRα7	1.18 ± 0.03	0.41 ± 0.07
nAChRα4	1.78 ± 0.20	1.06 ± 0.07
nAChRβ2	0.74 ± 0.07	0.97± 0.14

Data are reported as mean ± SEM. ** *p* < 0.01; *** *p* < 0.001.

**Table 6:** Gene expression (2^−ΔΔCt^) of IL-1β, TNFα, BuChE, AChE, nAChRα7, nAChRα4 and nAChRβ2 in the hippocampus of 4- and 8-month-aged SAMP8 mice in relation to the hippocampus samples from the age-matched SAMR1 mice.

	4-Month-Aged SAMP8	8-Month-Aged SAMP8
IL-1β	8.58 ± 0.92 ***	8.90 ± 1.04 ***
TNFα	1.46 ± 0.21 *	1.64 ± 0.13 ***
BuChE	15.54 ± 0.59 ***	17.35 ± 0.59 ***
AChE	1.16 ± 0.12	1.62 ± 0.17
nAChRα7	4.39 ± 0.41 ***	4.96 ± 0.34 *
nAChRα4	1.96 ± 0.28	4.03 ± 0.41 **
nAChRβ2	1.13 ± 0.16	1.34 ± 0.20

Data are reported as mean ± SEM. * *p* < 0.05; ** *p* < 0.01; *** *p* < 0.001.

**Table 7:** Gene expression (2^−ΔΔCt^) of IL-1β, TNFα, BuChE, AChE, nAChRα7, nAChRα4 and nAChRβ2 in the hippocampus of 4- and 8-month SAMP8 mice in relation to the composite brain samples from the same mice.

	4-Month-Aged SAMP8	8-Month-Aged SAMP8
IL-1β	3.03 ± 4.23 *	2.48 ± 2.74 ***
TNFα	2.70 ± 2.96 ***	2.27 ± 2.35 ***
BuChE	1.58 ± 1.71 *	2.38 ± 2.66 ***
AChE	1.08 ± 1.14	1.84 ± 1.98 **
nAChRα7	4.11 ± 4.80 ***	8.01 ± 1.49
nAChRα4	0.72 ± 0.77	0.98 ± 1.04
nAChRβ2	1.16 ± 1.33	2.12 ± 2.44 **

Data are reported as mean ± SEM. * *p* < 0.05; ** *p* < 0.01; *** *p* < 0.001.

**Table 8:** Primer pair sequences used in the study.

Gene	Mouse PCR Primer Pairs [5′-3′]	
	Forward	Revers
HPRT	TTGGATACAGGCCAGACTTTG	TGGCAACATCAACAGGACTC
BuChE	TAGCACAATGTGGCCTGTCT	ATTGCTCCAGCGATGAAATC
AChE	ATTTTGCCCGCACAGGGGAC	CGCCTCGTCCAGAGTATCGGT
nAChRα7	TGATTCCGTGCCCTTGATAG	GAATGATCCTGGTCCACTTAGG
nAChRα4	GTAGAAGGCGTCCAGTACATTG	AGATCATACCAGCCAACCATG
nAChRβ2	GCTTCATTGCGGACCATATG	CCAAAGACACAGACAAAGACAAAG
IL-1β	TTGACGGACCCCAAAAGATG	AGAAGGTGCTCATGTCCTCA
TNFα	TGGAGTCATTGCTCTGTGAAG	CCTGAGCCATAATCCCCTTTC

## Data Availability

Not applicable.

## Supplementary Materials

The following supporting information can be downloaded at: https://www.mdpi.com/article/10.3390/ijms232315097/s1.
