# Supplementary material for: Proteomic Signature and mRNA Expression in Hippocampus of SAMP8 and SAMR1 Mice during Aging
Source: Int J Mol Sci. 2022 Dec 1;23(23):15097. doi: 10.3390/ijms232315097 (PMC9740614; doi:10.3390/ijms232315097)
Supplement: Supplementary file 1 [file ijms-23-15097-s001.zip › ijms-2043249-supplementary.pdf]

Supplementary Material

Table S1: Hippocampal protein MS-identified in SAMR1 4 month aged.

AC: accession number, to identify the protein from SWISS PROT database; FV: fold variation,

	Protein name	Acronyms	AC	Theoretical Mr_pI	Experimental Mr_pI	*p*	PMF/ MS_2	FV
Up-regulated	Actin-related protein 2/3 complex subunit	**ARPC5**	Q9CPW4	16335_5.47	16901_5.1	0.004	4/1	0.3±0.02
Down-Regulated	Stathmin (1)	**STMN1**	P54227	17264_5.76	17158_5.6	$6.4x10^{-6}$	11/2	0.6±0.1
Down-Regulated	Stathmin (2)	**STMN1**	P54227	17264_5.76	17406_5.3	$7.5x10^{-7}$	11/2	1.1±0.08
Down-Regulated	Alpha-soluble NSF attachment protein	**SNAA**	Q9DB05	33624_5.30	26297_5.1	0.1	8/2	0.7±0.1
Down-Regulated	Inorganic pyrophosphatase	**IPYR**	Q9D819	33102_5.37	27560_5.3	0.004	14/-	1.7±0.2
Down-Regulated	Annexin A5	**ANXA5**	P48036	35787_4.83	27098_4.8	0.2	19/1	1.6±0.07
Down-Regulated	EF-hand domain-containing protein D2	**EFHD2**	Q9D8Y0	26775_5.01	26775_5.01	$5.3x10^{-5}$	20/2	1.6±0.08
Down-Regulated	Calbindin OS	**CALB1**	P12658	30203_4.71	23050_4.7	0.004	18/2	0.8±0.2
Down-Regulated	Malate dehydrogenase cytoplasmatic	**MDHC**	P14152	36659_6.16	27797_5.6	0.009	16/1	1.6±0.15
Down-Regulated	Microfibrillar-associated protein 3-like	**MFA3L**	Q9D3X9	45769_5.00	26337_6.1	0.1	9/-	0.9±0.17
Down-Regulated	Fatty acid-binding protein, heart	**FABPH**	P11404	14810_6.11	14560_5.4	$3.0x10^{-4}$	9/3	1.2±0.05
Down-Regulated	Superoxide dismutase	**SODC**	P08228	16104_6.02	16104_5.9	$6.8x10^{-4}$	4/2	1.0±0.08
Down-Regulated	Protein phosphatase 1 regulatory subunit 7	**PP1R7**	Q3UM45	41380_4.85	34021_4.9	0.01	12/-	0.4±0.1
Down-Regulated	Protein phosphatase 1 regulatory subunit 7	**PP1R7**	Q3UM45	41380_4.85	33971_4.9	0.02	13/2	0.4±0.1
Down-Regulated	40S ribosomal protein SA	**RSSA**	P14206	32931_4.80	31644_4.8	0.01	15/3	1.47±0.2
Down-Regulated	Dopachrome decarboxylase	**DOPD**	O35215	13183_6.09	14213_6.1	0.002	6/3	0.7±0.2

expression level degree. Mr2: relative molecular mass; PMF/ MS2: Peptide mass fingerprint/ Mass spectrometry. Statistical significance: $p<0.001$.

Table S2: Hippocampal protein MS-identified in SAMP8 4 month aged.

	Protein name	Acronyms	AC	Theoretical Mr_pI	Experimental Mr_pI	p	PMF/ MS_2	FV
Up-regulated	Kemitine reductase mu-crystallin	**CRYM**	O54983	33673_5.44	29128_5.2	$8.3x10^{-8}$	10/3	2.0±0.2
Up-regulated	Phosphoglycerate mutase 1	**PGAM1**	Q9DBJ1	28928_6.67	24391_6.3	$5.2x10^{-5}$	13/3	1.8±0.2
Up-regulated	V-type proton ATPase subunit B, brain isoform	**VATB2**	P62814	56857_5.57	37762_5.7	0.01	14/2	1.5±0.3
Up-regulated	Dual specificity protein phosphatase 3	**DUS3**	Q9D7X3	20687_6.07	20633_5.7	$7.6x10^{-4}$	8/3	0.4±0.2
Up-regulated	Malate dehydrogenase cytoplasmatic	**MDHC**	P14152	36659_6.16	28607_6.2	0.006	16/2	1.8±0.1
Down-regulated	Eukaryotic initiation factor 4A-I	**IF4A1**	P60843	46125_5.32	34942_5.3	0.007	20/3	2.0±0.05
Down-regulated	Stathmin	**STMN1**	P54227	17264_5.76	17158_5.6	$6.4x10^{-6}$	11/2	0.6±0.1
Down-regulated	Stathmin	**STMN1**	P54227	17264_5.76	17406_5.3	$7.5x10^{-7}$	11/2	1.1±0.08
Down-regulated	Superoxide dismutase	**SODC**	P08228	16104_6.02	16104_6.0	0.001	11/2	1.3±0.05
Newely costitutive	Pyridoxal kinase	**PDXK**	Q8K183	35278_5.88	28997_5.6	0.06	9/2	1.6±0.4

AC: accession number, to identify the protein from SWISS PROT database; FV: fold variation, expression level degree. Mr2: relative molecular mass; PMF/ MS2: Peptide mass fingerprint/ Mass spectrometry. Statistical significance: $p<0.001$.
